# Gendered digital housekeeping and invisible care labor in platformed higher education

**DOI:** 10.3389/fsoc.2026.1790117

**Published:** 2026-03-25

**Authors:** Xiang Li, Kenny S. L. Cheah, Yiing Siing Wong

**Affiliations:** 1 Institute for Advanced Studies, Universiti Malaya, Kuala Lumpur, Malaysia; 2 Faculty of Education, Universiti Malaya, Kuala Lumpur, Malaysia; 3 Faculty of Creative Arts, Universiti Malaya, Kuala Lumpur, Malaysia

**Keywords:** academic platformization, affective capitalism, digital care, ethics of care, gendered labor, governance of care, invisible work

## Abstract

As higher education continues to undergo rapid digital transformation - particularly since hybrid teaching became the routine during and after the pandemic; Learning Management Systems such as Canvas and Blackboard, communication tools like Teams and WhatsApp, and video platforms including Zoom and Tencent Meeting have become embedded in the everyday infrastructure of academic work. Much of the existing literature on platformed higher education focuses on macro-level processes such as datafication, managerial oversight, and neoliberal audit culture while far less attention has been paid to the embodied, routine practices that structure daily academic life. This article adopts a sociological perspective to examine how platform architectures generate fragmented forms of invisible care labor: routine technical maintenance, around-the-clock responsiveness to student queries, and screen-mediated emotional labor. Although essential to the functioning of digital teaching environments, these forms of labor remain largely unrecognized in formal evaluation systems and exhibit a distinctly gendered pattern. Women scholars disproportionately assume responsibility for repairing gaps in digital infrastructures, easing student anxiety, and sustaining online engagement. By analyzing how audit-oriented evaluation regimes fail to register this labor, the article illuminates the tension between metric-driven assessment and an ethics of care. Drawing on debates surrounding right-to-disconnect policies and gender-inclusive design, it proposes governance pathways that make digital care labor more visible and support more equitable and sustainable institutional arrangements.

## 1 Introduction

Over the past two decades, higher education systems worldwide have been reshaped by digital transformation. Technology is no longer simply a supplementary channel for delivering knowledge; it now constitutes a structural condition of higher education or academic life. This shift has altered the organization of academic labor, faculty–student relations, and institutional governance. The concept of the platformization of the university has been widely used to capture this broader trend. Universities increasingly operate through processes of data extraction, algorithmic management, and market-oriented logics that reorder educational priorities and redistribute resources (Tolmayer and Bedo, 2019; Komljenovic, 2021). This macro-level perspective has been essential for clarifying the political economy of contemporary higher education. Yet it often overlooks how digital platforms are actually encountered and managed in everyday academic practice. In particular, we know far less about how interface-level interactions reshape the temporality, spatial organization, and affective texture of academic work.

The present analysis shifts attention from platform capitalism as an abstract structural critique to the everyday practices mediated through interfaces. Platform capitalism refers to a mode of organizational restructuring in which digital platforms mediate institutional activities through data extraction, monetization logics, and algorithmic coordination. Within universities, platforms increasingly function as infrastructures of academic life rather than as neutral administrative tools. Drawing on platform and infrastructure studies (Plantin et al., 2018; van Dijck et al., 2018), this article treats platforms as connective systems that organize participation while embedding rules, defaults, and asymmetries that shape what users can do and how they relate to one another. Through notification systems, interface design, and interaction logics, platforms recalibrate the boundaries between presence and absence, work and non-work, responsiveness and silence. These micro-level transformations remain insufficiently theorized in discussions of higher education platformization.

At the same time, digital platforms have undeniably enabled new forms of institutional coordination and pedagogical flexibility. Learning management systems support centralized communication, continuity across dispersed settings, and structured distribution of resources. Data dashboards and interaction records can facilitate accountability and evidence-informed decision-making. For many students—particularly those balancing employment, caregiving, or geographical distance—digital mediation expands access. The appeal of platformization thus lies precisely in these affordances. Yet these same affordances also recalibrate expectations. Constant connectivity increases traceability and subtly intensifies norms of availability and responsiveness. What enables flexibility simultaneously redistributes maintenance and relational labor in ways that remain unevenly recognized.

As digital technologies become fully embedded in academic practice, higher education increasingly operates under conditions of boundarylessness, in which work expectations extends across time and space (Jansson et al., 2025). Email, messaging applications, and Learning Management Systems (LMS) platforms create an always-accessible network that stretches into domestic life, leisure and rest. Existing research suggests that the burdens associated with this expansion are unevenly distributed. The smooth functioning of digital systems often depends on additional, fragmented forms of labor that remain institutionally invisible. These include responding to students outside formal hours, reorganizing materials for mobile access, and managing anxiety during technical disruptions (Sciannamblo, 2024). This article conceptualizes such practices as digital invisible care labor: an ongoing maintenance, coordination, and affective work required to sustain instructional continuity and relational stability within platform-mediated environments. This labor does not arise solely from individual goodwill. It is continuously generated and intensified by structural features embedded in interface design and default connectivity settings. Fragmented message streams, expectations of immediate feedback, and visible interaction traces render responsiveness normative, while silence risks being interpreted as neglect or professional failure. Building on prior discussions of academic housekeeping and relational service work, the analysis extends this line of inquiry by examining how digital platforms restructure the temporal and affective intensity of such labor. Compared with pre-digital coordination, digital invisible care labor is more tightly woven into platform architectures. Persistent notifications, cross-device access, and quantifiable traces institutionalize expectations of continuous availability and heighten pressures of emotional regulation.

Existing empirical research in sociology and gender studies points to persistent gender disparities in care responsibilities and emotional labor. In this context, emotional labor involves managing one’s own feelings and performing care-oriented affective work to meet institutional and interpersonal expectations. Women disproportionately perform these forms of labor in both domestic and professional settings (Docka-Filipek et al., 2023). When care work is mediated through digital platforms, these gendered patterns may be reproduced and, in some institutional contexts, amplified through platform architectures and normative expectations of availability. Studies have shown that women faculty are more frequently expected to be approachable, responsive, and patient, and may face higher levels of routine digital demands and informal student requests (El-Alayli et al., 2018; Guarino and Borden, 2017). By contrast, men faculty may be better positioned to disengage from routine digital housekeeping and concentrate on activities more directly rewarded by indicator-driven evaluation systems. Here, digital housekeeping refers to the routine, often low-visibility maintenance tasks required to keep digital teaching platforms functional, organized, and responsive in everyday academic practice. This article builds on a gendered analytical lens. It examines the production and allocation of invisible digital care labor through platform interfaces and analyzes how quantification-centered evaluation regimes overlook its value. It also considers governance-oriented responses, including right to disconnect policies and more inclusive platform design.

A conceptual analysis is undertaken here that synthesizes insights from feminist sociology, platform studies, and higher education research. Its primary contributions are threefold. Conceptually, it develops the notion of digital invisible care labor to capture the gendered, affective, and infrastructural dimensions of academic work that remain obscured in dominant accounts of platformization. Analytically, it links interface design and audit culture to the uneven distribution and institutional invisibility of care labor in platformed universities. From a governance perspective, it outlines policy and design-oriented interventions. These interventions address digital overwork and inequitable labor allocation, including the right to disconnect and principles of inclusive platform design. The right to disconnect affirms employees’ entitlement to disengage from work-related digital communication outside formally designated working hours.

The discussion centers on higher education systems in which digital platforms are deeply embedded in teaching, evaluation, and academic communication. While many illustrative examples and e9mpirical references draw from Anglophone and Global North contexts, the argument is not limited to any single national system. Rather, it aims to offer an analytically portable framework for understanding how platformization and gendered care labor intersect across different institutional and policy environments. Variations in national regulation, levels of digital infrastructure, and institutional type are acknowledged as important factors shaping these dynamics and are discussed as directions for future comparative research.

## 2 Digital housekeeping inside the ivory tower

Digital housekeeping has become a routine yet remains largely unexamined component of contemporary academic work. Digital housekeeping first emerged specifically from family sociology and other related research to describe the ongoing maintenance required to keep connected homes and digital infrastructures functioning, managing passwords, updating software, troubleshooting devices (Teigen, 2024). Although essential to everyday digital convenience, such work is often treated as trivial and presumed to occur automatically. Within households, it is frequently gendered (Peng, 2022) when it comes to division of labor. A similar logic now operates within universities classrooms settings. Thus, with the advent LMS platforms such as Canvas, Blackboard, and Moodle have become central to teaching, faculty members have increasingly assumed responsibilities akin to frontline system operators. Faculty have ceased from being just instructors. They have been refreshed to configure course shells, manage permissions, repair broken links, and address recurring technical issues. Rarely has this shift been framed as formal job redesign, rather these responsibilities are instead folded quietly into institutional expectations and default platform settings.

Digital platforms are not neutral tools. Their design logics shape labor. In widely used LMS environments (as with Canvas and Blackboard in particular), interface structures often reflect managerial priorities: standardization, modularization, traceability, with less attention to the instructor experience. For instructors, teaching-related work becomes decomposed into numerous small acts of clicking, navigating nested menus, verifying settings. Beyond the general standardization, specific interface design choices also actively configure instructors’ workload. Many LMS platforms rely on default real time notification settings that immediately alert instructors to submissions, comments, or attend to minor student interactions, compressing response windows unless manually reconfigured. This is further compounded by often complex hierarchical permission and role based access structures, requiring instructors to repeatedly verify visibility settings to avoid accidental exposure or restricted access. Template driven course shells may enforce predefined module structures that privilege administrative traceability over pedagogical flexibility, compelling instructors to adapt their teaching design to platform logics rather than the reverse. Additionally, activity logs and analytics dashboards preserve traceability, render response times and interaction frequencies measurable and implicitly comparable. In this way, routine coordination becomes performance-visible labor.

Usability research on Learning Management Systems have demonstrated that non-intuitive interfaces impose not only substantial learning cost but also more cognitive load. Faculty must memorize action sequences and devote disproportionate time to routine tasks (Bousbahi and Alrazgan, 2015). Platform migrations further intensify this burden. When institutions upgrade or switch systems, instructors must relearn operational logics and reconstruct course materials. Studies of LMS migration highlight that such transitions generate hidden time costs, additional troubleshooting, and expanded demands on instructors beyond their teaching load. This is because migration is often framed as administrative modernization rather than as labor-intensive change. In practice, it involves fragmented hours spent converting formats, repairing links, redesigning navigation structures, and troubleshooting errors. This work is cognitive, emotional, and often accompanied by frustration and anxiety over potential teaching disruption.

Like other physical infrastructures, digital housekeeping becomes visible primarily when it fails (Whiting and Symon, 2020). When an online course runs smoothly, the labor behind functioning links, correct permissions, and accessible materials remains unnoticed. Yet minor breakdowns, however, can trigger immediate doubts about instructor’s competence or professionalism but gains wider visibility. This asymmetry produces a high-risk, low-reward form of labor in digital housekeeping. In earlier academic divisions of labor, teaching assistants or administrative staff might share some of this work which is no longer true today. Under cost-cutting and self-service trends associated with neoliberal governance, technical and administrative tasks have increasingly shifted to frontline instructors, particularly junior and contingent faculty (Bou Reslan and El Hokayem, 2023). The result is intensified technostress, reduced time for research and teaching preparation. Empirical studies link these accumulated demands to occupational strain. Technostress in higher education, technostress is conceptualized as a multidimensional person–environment misfit; shaped by institutional ICT requirements that exceed available time, skill and support (Wang and Li, 2019) with implications for work related functioning and well-being. Evidence from Norwegian academia similarly associates digital stressors, including overload, complexity, and unreliability, are associated with emotional exhaustion among higher education employees. These outcomes proofs that digitally intensified work systems can generate sustained strain rather than episodic inconvenience (Sevic et al., 2025). In another vein, a longitudinal research further suggests that, digitally intensified work environments affect job satisfaction and long-term psychological responses particularly how teachers evaluate their work experience (Nascimento et al., 2025).

## 3 The digital extension of care labor

### 3.1 Digital emotional labor behind the screen

If digital housekeeping sustains systems, screen digital emotional labor sustains people. Teaching is a relational practice that involves care, encouragement, and emotional regulation (also termed affect regulation). Here, affect regulation denotes the ongoing management and modulation of emotions in order to sustain pedagogical relationships and classroom stability. When interaction moves onto digital platforms, it becomes distorted in several ways. First, in digital environments, these demands shift in form and intensity. For instance, mediated channels such as email and instant messaging lack many non-verbal cues. Instructors must do more interpretive or infer emotion from brief messages and carefully calibrate tone to convey empathy. Emojis, punctuation, and phrasing become tools of relational signaling. Second, remote and hybrid contexts often amplify student vulnerability. Students in this settings often report loneliness, uncertainty, and anxiety, thereby seeking reassurance as much as content clarification. Platforms such as Zoom classes further complicate relational dynamics. Teaching to muted microphones and blank screens deprives instructors of immediate feedback. Sustaining such classroom energy becomes performative and exhausting, an experience frequently described as Zoom fatigue (Bellas, 1999; Nesher Shoshan and Wehrt, 2022).

### 3.2 Always-on expectations and the colonization of time

Instant messaging tools such as Slack, Teams, WeChat, and WhatsApp, together with and LMS notifications create implicit expectations of perpetual availability. Platform architectures have also shortened perceived question and response windows. Students influenced by these digital consumer cultures may approach instructors as on-demand support providers as well as expect near instant gratification. Research on chronemic in Instructor-Student expectations suggests that slower email responses can reduce perceived credibility and relational closeness; such that responses within approximately one day are often treated as baseline acceptability (Tatum, 2021). In practice, these expectations extend into evenings and weekends. When students do not receive replies outside formal working hours, some may experience anxiety or dissatisfaction and express this in course evaluations. Instructors may remain hypervigilant, fearing missed messages. Empirical evidence in higher education indicates that such digitally intensified vigilance and interruption patterns are associated with emotional exhaustion, consistent with concerns that weakened recovery and persistent connectivity can carry measurable costs for occupational wellbeing (Sevic et al., 2025). This creates an environment wherein, academic work becomes spatially unbounded, extending into domestic space or private time; turning bedrooms, dining tables, and travel spaces into potential workplaces. Without institutional boundaries, the workday lacks closure as long as the phone remains within reach. More still, it renders psychological detachment difficult, undermining recovery and interrupts sustained research concentration.

### 3.3 Pandemic acceleration and compassion fatigue

The COVID-19 pandemic intensified these dynamics. During emergency remote teaching, instructors were urged to demonstrate empathy and flexibility support students affected by the illness, mental health and financial crisis (Riley et al., 2021). Extension requests such like special arrangements, and crisis communication and sometimes functioning as temporary counselors dramatically expanded emotional demands. Although in the post pandemic period emergency conditions have receded, many expectations persist within institutionalized hybrid systems remain central. This ongoing digital acceleration pushes instructors to maintain high-intensity online emotional supply alongside traditional teaching responsibilities, contributes to compassion fatigue and burnout.

## 4 Gendered digital division of labor

More critical than the intensification of digital demands is the question of how these demands are allocated within academic institutions. Digital housekeeping and emotional labor is not evenly distributed. Research across sociology and gender studies consistently identifies gendered patterns in emotional and service work. In domestic contexts, women perform most housework and caregiving. Students often hold different expectations of women and men instructors. Women are more likely to be perceived as approachable and nurturing; men as authoritative and expert (Bellas, 1999; El-Alayli et al., 2018). These expectations shape patterns of request and in this case women faculty frequently receive more emotional support or disclosures, extension negotiations, and technical inquiries. When LMS problems occur, students may be more likely to seek step by step guidance from women instructors, while treating men instructors as recipients of problem reports rather than care work.

Sociologists have described women’s disproportionate service burdens in academic institutions as academic housekeeping or hidden service (Lopes and de Camargo Santos, 2025). Even after controlling for rank and field, women perform more institutional service (Guarino and Borden, 2017). The findings have shown that women are as most likely to carry tasks that include organizing meetings, writing minutes, maintaining relationships, and supporting student wellbeing than men. In digital environments, this extends into what might be termed digital nannying: refining LMS layouts, composing detailed announcements, sustaining discussion forums and a welcoming online environment. Such work represents not mere esthetic preference but relational investment, i.e., a form of emotional investment and work inclusion.

Women instructors may also experience deeper emotional entanglement. This is because students sometimes disclose technical struggles or personal difficulties. In these situations, women can face stronger pressure to remain available and to provide extra tutoring or emotional support and refusal often carries differential risk. Women who set boundaries may be labeled unresponsive or uncaring (Castelao-Huerta, 2024). Anticipating relational consequences, they may engage in preventative maintenance fine-grained technical corrections to avoid breakdowns. Over time, these dynamics create self-reinforcing cycles. On the contrary, stereotypes often portray men as more technical.

Although comprehensive large-scale statistics remain limited, qualitative studies and smaller quantitative analyses consistently point to these gendered differences. However, existing empirical evidence remains uneven across national contexts and institutional types, and systematic comparative analyses are still limited. Further research is needed to examine how these gendered patterns intersect with race, class, employment status, and institutional hierarchy in different higher education systems. For example, student reports during the early COVID-19 period indicated that women faculty were perceived as more supportive and accommodating, which often implied that they carried greater care labor burdens (Docka-Filipek et al., 2023). This gendered allocation cannot be simply attributed to differences in technical competence. Although some studies suggest that gender gaps in digital learning-related self-efficacy and attitudes are narrowing, expectations that women should be more supportive persist. At the same time, women often bear a heavier emotional and relational load in technology-mediated teaching. Research on emotional labor in universities during COVID-19 similarly highlights gendered patterns in care work and exhaustion (Casey et al., 2025). When women scholars confront the technical complexity of an LMS, the strain is therefore not limited to operational difficulty. It also includes the anxiety that a technical failure may undermine the trust and rapport they have worked to build with students, which can in turn generate further demands for support and accommodation. Together, these dynamics form a self-reinforcing cycle. By contrast, men who set boundaries more effectively can concentrate on research outputs that carry more weight in evaluation. As a result, they may advance faster. Digital platforms therefore do not automatically reduce gender gaps. Rather they intensify academic gender inequality through subtle mechanisms than neutralizing.

Gender serves as the primary analytic lens in this section, yet the mechanisms described above extend beyond gender alone. Digital invisible care labor is embedded within layered institutional hierarchies that shape both expectations and the capacity to refuse them. Structural position within the academic field conditions how platform-based care demands are experienced and negotiated. For example, early-career scholars and contingent faculty may encounter intensified pressures of responsiveness not only because of gendered expectations, but because their institutional authority is limited and their employment security fragile. Similarly, academics in teaching-intensive or lower-status institutions may face stronger normative expectations of availability as a condition of professional legitimacy. These intersecting structural positions do not simply add to gender; rather, they recalibrate how gendered expectations operate within specific organizational contexts. These patterns intersect with career stage, employment precarity, and institutional hierarchy. Early-career and contingent faculty often face amplified responsiveness pressures due to limited authority and security. Digital invisible care labor thus emerges through layered interactions among gender norms, precarity, and evaluative regimes.

## 5 Blind spots in evaluation systems

Neoliberal logics now permeate contemporary higher education and shape how academic value is defined and assessed. Metric-driven evaluation systems prioritize ranked indicators: H-index, citation counts, impact factors, grant income, student evaluations. Teaching quality is reduced to dashboards and satisfaction scores. University management relies on these metrics for performance assessment, promotion, and resource allocation. Scholars adapt by quantifying their academic selves, curating data profiles on platforms such as ResearchGate and Google Scholar, and engaging in forms of gamification (Hammarfelt et al., 2016). Digital invisible care labor remains largely unregistered. This quantified logic also penetrates teaching evaluation, where teaching quality is simplified into LMS activity dashboards and end of term satisfaction scores. Yet evaluation systems are largely blind to digital invisible care labor. This blindness appears in three interrelated ways.

First, LMS backend data can be misleading. Platforms can record logins, clicks, time online, and message counts, but they cannot measure care. Data conspicuously lack context. A platform can log that an instructor replied to an email, but it cannot capture the emotional reassurance embedded in the reply. It can log 3 h spent inside an LMS, but it cannot distinguish creative course design from time lost fighting poorly designed formatting or broken interfaces. Datafication here strips labor of context. Second, qualitative indicators are missing. In many performance systems, only teaching hours and research outputs are formal categories. Performance systems often lack explicit workload categories for digital maintenance and emotional support. As a result, invisible care becomes background noise that is expected but not rewarded. Third, a reverse punishment mechanism emerges. Women instructors who invest heavily in digital care may be judged as low performing because research outputs decline. If they reduce care to protect research time, they may face worse student evaluations because they are perceived as unresponsive or insufficiently supportive. This is a governance trap produced by audit culture, in which the measurable is treated as the meaningful. Classic analyses of audit culture show how measurement systems reshape behavior by privileging what is countable and marginalizing what is ethically and relationally central.

Digital platforms are not merely passive systems that record data and invisible care becomes assumed background labor. They operate as governance technologies in their own right. When complex academic labor is compressed into visual dashboards, platforms produce a trap of visibility in which what can be counted comes to stand in for what matters. Tasks that are readily quantifiable, such as the number of assignments graded or the frequency of posts, become disproportionately emphasized. Rather a reverse punishment dynamics have emerged whereby those investing heavily in care risk research penalties. In other words, forms of labor that resist quantification, such as building trust, providing emotional support, and repairing technical breakdowns, are systematically pushed out of the institutional horizon of value. This evaluative blind spot contributes to the alienation of academic labor, because scholars are forced into a zero sum struggle between being responsible to students through sustained care and being responsible to indicators through measurable output. For women scholars, gender norms and expectations often make it harder to relinquish the former, which places them at a disadvantage in career competition. This alienation does not only harms individual academic development, it also erodes the core values of higher education, namely the genuine and caring intellectual connection between people. When measurable output becomes synonymous with value, relational labor recedes from institutional recognition.

## 6 Toward care-oriented governance

Addressing these dynamics requires structural responses; crisis of such magnitude cannot rely on individual time management or self-care alone. It is a systemic problem of organizational governance and technology design. Higher education needs policy, technical design, and cultural change that rebalances quantification with an ethics of care.

### 6.1 Institutional pathways

Right-to-disconnect policies offer one model: Universities should adopt clear policies that protect staff rights to be offline outside working hours without penalty. This is not only a legal compliance issue; it is central to reshaping academic labor ethics. For example, the Right to Disconnect Policy at Trent University affirms staff entitlement to disengage from work-related communication outside designated hours. It also emphasizes mutual obligations not to intrude on colleagues’ nonwork time (Trent University, 2022). Organizational guidance on implementing right to disconnect policies similarly stresses practical norms, leadership support, and clear expectations that responsiveness speed should not be treated as a measure of commitment (Lancaster University Work Foundation, 2021).

Effective implementation requires both communication norms and concrete technical measures. Communication norms specify normal work hours and set reasonable response windows. Technical measures delay non-urgent notifications outside work hours. Leadership endorsement can also reduce peer pressure for constant responsiveness. Universities should also reform workload allocation models so that digital maintenance and emotional labor become visible and countable in fair ways (Wang, 2025; Ministry of Education, 2025). Workload models should explicitly recognize digital maintenance and student care. Feminist analyses show that nominally neutral workload systems often reproduce inequality unless care labor is formally acknowledged (Steinþórsdóttir et al., 2021). Hiring instructional technologists can reduce routine digital burdens. Institutions can add recognized categories such as digital infrastructure maintenance and student support and care, and they can give these categories meaningful weight in annual review. They can also reduce low-value digital chores by hiring instructional technologists. These technologists handle routine LMS migration, formatting, and technical setup.

### 6.2 Technical pathways

LMS design should prioritize humane workflows: LMS procurement and development should move beyond managerial logics toward designs that support humane teaching experience. Simplified navigation, reduced cognitive load, richer feedback modalities, and built-in boundary-setting tools can mitigate strain instead of requiring instructors to navigate layered menus and many settings for routine tasks. They can also add functions that support emotional expression, such as richer feedback options and easier voice or video responses, reducing misinterpretation in text only communication. Boundary setting features should be built into core platform functions so instructors can set office hours, control response channels, and pause notifications in ways that structurally support disconnection.

Feminist human-computer interaction calls for centering marginalized users, value subjective experience, and challenging assumptions of technical neutrality (Bardzell, 2010). Universities should conduct usability and equity testing before adopting new platforms. Gender-aware usability testing can identify design features that externalize hidden labor. Therefore, testing should include women faculty, contingent instructors, and other groups since these groups may face amplified burdens. This testing should use gender-aware sampling and include women faculty, contingent instructors, and other groups due the fact that these groups may face amplified burdens. The aim is not only to attain usability, but also identifying design defects that externalize hidden labor onto those already carrying disproportionate care work.

### 6.3 Cultural pathways

Finally, universities should cultivate cultures that resist digital acceleration. Slow computing and slow technology perspectives emphasize design and practice that protect reflection, pacing, and mental rest rather than constant reaction (Hallnäs and Redström, 2001). Academic communities can normalize response windows such as 48 h and create buffers to protect uninterrupted work time. They can also establish collective care networks. In these networks, responsibility for student support is shared institutionally rather than individualized. Such changes require both top down leadership signals and bottom up agreement among faculty.

## 7 Discussion and governance implications

Digital platforms enhance efficiency while generating uneven invisible burdens. Placing the central concepts of this article within broader debates helps clarify its theoretical contribution. Learning management systems and always-on messaging tools may enhance efficiency, yet they also generate substantial invisible burdens, including routine maintenance, emotional reassurance, and expectations of constant presence. These burdens accumulate disproportionately among those positioned as care providers while metric-driven governance remains largely blind to them. By privileging measurable outputs as primary indicators of academic value, these systems may further intensify the conditions under which such labor accumulates.

Stratified governance environments within higher education further shape these dynamics. The accumulation of digital invisible care labor is not uniform, as institutional authority, evaluative regimes, and employment arrangements are themselves unevenly distributed. Governance systems that rely heavily on quantified performance indicators often place greater pressure on those whose professional legitimacy depends on demonstrable responsiveness. Early-career and precariously employed academics operate under heightened scrutiny, where responsiveness becomes tied to professional survival. In such contexts, expectations of digital availability become embedded within institutional survival strategies.

These observations carry implications for governance, platform design, and academic culture. The intensification of digital invisible care labor cannot be addressed through calls for individual resilience alone; it requires structural and institutional change. More fundamentally, if universities are to retain the possibility of remaining spaces for reflection and care, it becomes necessary to question the dominance of metric-driven evaluative logics that increasingly shape academic work.

This analysis has certain limitations. As a conceptual inquiry, it does not offer new empirical evidence on how digital care labor is distributed across different institutional settings. Regulatory regimes, platform infrastructures, and labor arrangements vary considerably, and these differences are likely to shape the dynamics outlined above in distinct ways. Such limitations point toward the need for comparative research that examines how digital invisible care labor is experienced, organized, and governed across diverse institutional contexts.

## 8 Conclusion

Digital platforms do not simply increase workload; they reshape the conditions under which academic labor is organized and becomes visible, valued, and rewarded. Digital invisible care labor should not be understood simply as an overlooked burden. It reveals how particular forms of labor are continually produced within the governance arrangements of the platformised university. Notification systems, visibility norms, and metric-based evaluation frameworks together generate structural expectations of constant responsiveness. What appears as individual responsiveness is thus repositioned as a structurally embedded role, shaped through interface design and reinforced by audit-oriented institutional arrangements. The central issue is not merely who undertakes more labor, but how institutional terrains are configured so that such labor is persistently and unevenly required.

This institutional terrain emerges through the interplay of platform architectures, governance regimes, and academic cultures. Digital invisible care labor reveals how institutional arrangements produce and normalize particular forms of labor. A default notification setting, once repeatedly enacted, can gradually settle into institutional norm; such norms then crystallize into shared expectations before returning to individuals in the form of personal responsibility. Within this recursive process, care labor shifts from discretionary engagement to a default obligation, unevenly distributed yet widely naturalized.

Treating digital platforms as neutral tools of efficiency is no longer sufficient for understanding contemporary transformations in academic labor relations. Platforms function simultaneously as infrastructures and governance mechanisms, shaping what becomes visible or obscured, valued or disregarded. The persistence of gendered inequalities within academic careers is sustained not only by longstanding institutional structures but also by interfaces that normalize continuous availability, by metrics that fail to register affective labor, and by evaluative expectations shaped around response speed. Without sustained attention to these dynamics, efforts toward gender equity risk remaining confined to individual adjustment rather than institutional transformation. The concept of digital invisible care labor offers a way to trace how expectations circulate between technological systems and governance regimes, revealing forms of power that remain embedded yet insufficiently articulated within the platformized university.
